# Parapapillary choroidal microvasculature dropout in eyes with primary open-angle glaucoma

**DOI:** 10.1038/s41598-023-48102-8

**Published:** 2023-11-23

**Authors:** Ryoko Igarashi, Shun Ochiai, Tadamichi Akagi, Daiki Miyamoto, Yuta Sakaue, Ryu Iikawa, Takeo Fukuchi

**Affiliations:** Department of Ophthalmology and Visual Science, Graduate School of Medical and Dental Sciences, Niigata University, 1-757 Asahimachido-ri, Chuo-ku, Niigata, 951-8510 Japan

**Keywords:** Optic nerve diseases, Outcomes research

## Abstract

The purpose of this study was to evaluate how various parameters are related to microvasculature dropout (MvD) area measured using optical coherence tomography angiography (OCTA). We measured the area of MvD in 55 patients with primary open-angle glaucoma (POAG). Using OCTA, MvD area and peripapillary choroidal atrophy (PPA) area were assessed in a 4.5 mm × 4.5 mm region. The following were examined: circumpapillary nerve fiber layer (cpRNFL) thickness, optic disc area, optic disc cupping area, optic disc rim area, Humphrey Field Analyzer (HFA) 24/10–2 mean deviation (MD), and pattern standard deviation (PSD). The relationship between MvD area and each parameter was evaluated using Spearman’s rank correlation coefficient analysis. Mean MvD area and PPA area were 0.18 ± 0.17 mm^2^ and 1.13 ± 0.72 mm^2^, respectively. MvD area was significantly correlated with optic disc rim area (*p* = 0.0017), cpRNFL (*p* = 0.0027), HFA 24/10–2 MD, and PSD (*p* < 0.001). In eyes with POAG, MvD area indicates the severity of glaucoma, which might be associated with structural changes in the peripapillary vasculature around the optic disc.

Microvasculature dropout (MvD) refers to the loss of microvessels in the deep layers around the optic disc. It is often found in glaucoma^1–3^ and glaucoma with myopia^4–6^. In 2017, Lee et al. reported that MvD findings in the parapapillary choroid with OCT-angiography (OCTA) correspond to findings with ICG angiography^7^. This result suggests that MvD reflects the lack of blood flow in the deep layers. In addition, MvD has been observed at the site of optic neuropathy in primary open-angle glaucoma (POAG). Macular and optic nerve head superficial vessel densities are related to the progression of glaucomatous optic neuropathy^8^. Previous reports have shown that superficial blood flow is associated with thinning of the inner retinal layer and is a predictor of glaucoma progression^8–10^. In addition, deep blood flow loss can be observed with peripapillary atrophy in glaucoma, but these findings are highly correlated with thinning of the retinal nerve fiber layer and visual field disorder^11,12^. These findings are also related to paracentral visual field disorder^13^ and subsequent progression of glaucoma^14^. Furthermore, some studies have shown that peripapillary choroidal MvD is closely associated with intrapapillary circulatory disorders^15,16^.

We previously analyzed the relationships between structural indices such as surface blood vessel density and inner retinal layer thickness of the optic disc circumference^17^ or macula^18^ in eyes with glaucoma, functional indices such as central visual field sensitivity, and patients’ clinical characteristics. We concluded that a decrease in vascular density in the foveal avascular zone and peripapillary site is correlated with inner retinal layer thickness and central visual field impairment.

Many reports have described parapapillary MvD and glaucoma parameters, but the relationship between central 10° visual field and MvD has not been fully investigated. Therefore, to clarify the relationship between the structure and function of the central 10° visual field, which is important for visual acuity and vision quality in glaucoma, we investigated MvD area in patients with POAG. We evaluated the association between MvD area with each parameter to investigate its relationship to MvD using OCTA.

## Methods

This study was approved by the ethics committee of Niigata University Medical and Dental Hospital (no. 2019-0055). The study was conducted in accordance with the Declaration of Helsinki. Informed consent was obtained from all patients, their legal guardians, or both.

### Participants and examinations

This study included 55 eyes (29 right eyes and 26 left eyes) with POAG in 55 patients (29 men and 26 women) who underwent spectral-domain optical coherence tomography (SD-OCT) at Niigata University Medical and Dental Hospital between February 2018 and September 2021 to produce clear OCTA images of the papillary area. Mean age was 60.69 ± 12.18 years, mean spherical equivalent was − 3.91 ± 2.96 diopters (D), mean Humphrey field analyzer (HFA)10–2 mean deviation (MD) was − 10.03 ± 6.97 dB, and mean HFA24-2 MD was − 10.96 ± 6.37 dB (Table 1).

**Table 1:** Baseline characteristics of the study patients.

	POAG (n = 55)
Age (years)	60.69 ± 12.18
Sex (male/female)	29/26
SE (D)	− 3.91 ± 2.96
Laterality (right/left)	28/27
IOP (mmHg)	14.18 ± 2.45
10–2 MD (dB)	− 10.03 ± 6.97
24–2 MD (dB)	− 10.96 ± 6.37
No IOP-lowering medication (PG/β-blocker/other)	52/38/54
Systemic condition (hypertension/diabetes)	3/3
MvD area (mm^2^)	0.18 ± 0.17
Peripapillary chorioretinal atrophy area (mm^2^)	1.13 ± 0.72
Corneal thickness (μm)	524.15 ± 37.84
Axial length (mm)	25.29 ± 1.37
Optic disc rim area (mm^2^)	0.64 ± 0.19
Optic disc area (mm^2^)	1.89 ± 0.4
Optic disc cupping volume (mm^3^)	0.54 ± 0.23
cpRNFL thickness (μm)	64.62 ± 9.59
Decimal VA	1.12 ± 0.2
logMAR	− 0.03 ± 0.16

*POAG* primary open-angle glaucoma; *SE* spherical error; *IOP* intraocular pressure; *MD* mean deviation; *PG* prostaglandin; *MvD* microvasculature dropout; *cpRNFL* circumpapillary retinal nerve fiber layer; *VA* visual acuity; *logMAR* logarithm of Minimum Angle of Resolution.

All patients underwent common ophthalmologic tests, including best-corrected visual acuity, refraction, corneal curvature, angle, intraocular pressure based on applanation tonometry, and visual field testing.

### Diagnosis of POAG

Glaucoma was diagnosed by at least two glaucoma specialists. We referred to the fifth edition of the Japan Glaucoma Society Guidelines for Glaucoma^19^. The diagnostic criteria for POAG are as follows: (1) open-normal appearing anterior chamber angle and (2) glaucomatous visual field changes corresponding to glaucomatous optic disc changes, including retinal nerve fiber layer defects. Glaucomatous visual field changes met at least one of the following criteria: (1) three abnormal points, with a probability of being normal of *p* < 5% and one point with a pattern deviation of *p* < 1%, (2) a pattern SD or CPSD of *p* < 5%, or 3) values outside the normal limits in the glaucoma hemifield test. Glaucomatous optic neuropathy included at least one of the following: (1) vertical elongation of the optic cup, with an associated decrease in neuroretinal rim width (vertical cup-to-disc ratio ≥ 0.7, asymmetry ≥ 0.2); (2) notching of the neuroretinal rim; (3) disc hemorrhage; (4) parapapillary atrophy; (5) baring of the circumlinear vessel; and (6) exclusion of diseases that might cause glaucomatous changes in the optic disc. Inclusion criteria were: (1) POAG, (2) use of the Swedish Interactive Thresholding Algorithm (SITA) to acquire reliable test results (< 20% poor fixation and < 15% false-positive and false-negative rates), and (3) clear images obtained with Cirrus HD-OCT5000: Angiography 4.5 mm × 4.5 mm and Optic Cube 300 × 300, (4) signal strength index ≥ 7, and high-quality images were available for analysis.

Visual field was measured with a Humphrey Field Analyzer II (Carl Zeiss Meditec, Dublin, CA, USA) using the HFA central 10–2 and 24–2 SITA standard program test grid patterns. Only reliable test resultswere analyzed. Glaucomatous visual field defects were judged in accordance with Anderson and Patella’s standard^20^. When both eyes of a patient could have been included, the eye with worse visual field defects was included. Exclusion criteria were refractive values with astigmatism ≥ + 2D or ≤ − 2D, spherical equivalent ≤ − 8D, history of intraocular surgery, and history of eye disorders adversely affecting visual acuity or the visual field, including vascular occlusive diseases. The baseline characteristics of the study patients are shown in Table 1.

### Measurement and determination of the presence of MvD

**Figure 1:**
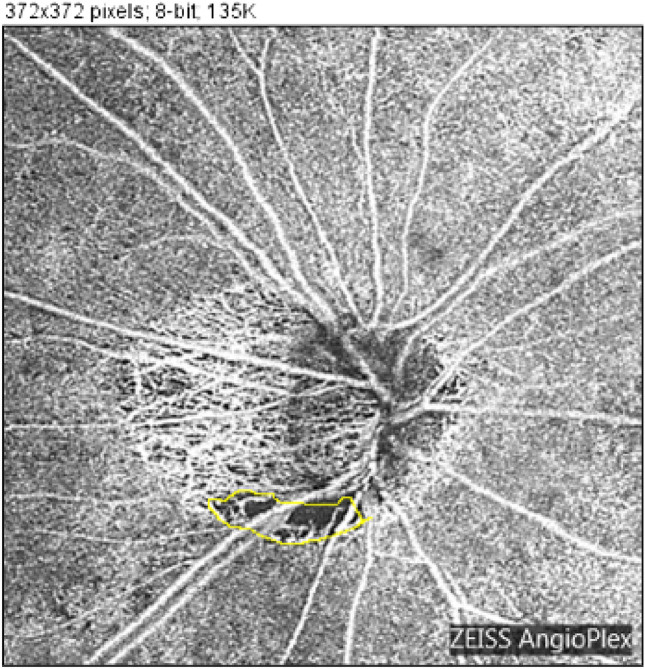
Manual measurement and computation of microvasculature dropout. Optical coherence tomography angiography (OCTA) was performed using HD-OCT (Cirrus HD-OCT5000 with AngioPlex OCT Angiography). Optic nerve angiography images were obtained using a 4.5 mm × 4.5 mm scan pattern. Images with signal strength index (an index of image reliability) ≤ 50 were excluded. Microvasculature dropout (MvD) area was identified manually using ImageJ analysis software (yellow line).

1. *Determination of the presence of MvD*: Peripapillary choroidal atrophy (PPA) was defined as deep choroidal atrophy around the optic disc. Inside the area of PPA, a defect of the microvascular shadow was defined as MvD (Fig. 1).
2. *Measurement of MvD*: Two examiners independently measured MvD area in OCTA images defined in the previous section using ImageJ analysis software (ImageJ, United States National Institutes of Health)^21,22^. OCTA was performed using an HD-OCT (Cirrus HD-OCT5000 with AngioPlex OCT Angiography; Carl Zeiss Meditec). During optic nerve angiography, a 4.5 mm × 4.5 mm scan pattern was used to obtain images (Fig. 1). Images with signal strength index (an index of the reliability of images) ≤ 50 were excluded. MvD area was identified manually using ImageJ analysis software (Fig. 1, yellow line). MvD identification was performed at least once by each orthoptist (S.O. and D.M.), for a minimum of two measurements in total. They determined the final measurement values by averaging all measurements for analysis. From these images, the selected area was regarded as the MvD area and further analyzed. The first author reviewed all OCT images with an orthoptist (S.O.).

### Statistical analysis

Relationships between MvD area and functional indices (measured sensitivity threshold (HFA10-2/24-2) MD and foveal threshold) were analyzed. Optic disc circumpapillary retinal nerve fiber layer (cpRNFL) thickness, PPA area, total optic disc area, optic disc rim area, and disc cupping area, all of which were determined with OCT, were considered structural indices. Age, spherical error (SE), and central corneal thickness (CCT), were considered clinical characteristics.

Spearman’s rank correlation coefficients were used to assess correlations between MvD area and functional and structural indices. We investigated these three types of parameters and their relationship to MvD area. For multivariable regression analysis, the forced entry method was performed with MvD area as the dependent variable; age, axial length, intraocular pressure (IOP), mean cpRNFL thickness, HFA 10-2/24-2 MD, and PPA area were included as independent variables. *p* < 0.05 was considered significant. Statistical analyses were performed with IBM SPSS Statistics for Windows, version 25.0 (IBM Corp., Armonk, NY).

## Results

### Intraobserver and interobserver reproducibility of MvD measurements

The intraclass correlation coefficients (ICCs) calculated using SPSS software were used to determine reproducibility as well as interobserver and intraobserver reliability of MvD measurements (Table 2). The intraobserver ICC for MvD was 0.983 (*p* < 0.001). The interobserver ICC (ICC_mean_) was 0.993 (*p* < 0.001) (Table 2).

**Table 2:** Intraobserver and interobserver reproducibility of MvD area measurements.

	ICC (mean)	
	r	*p* Value
Intraobserver	0.983	< 0.001
Interobserver	0.993	< 0.001

ICCs were determined with SPSS.

*ICC* intraclass correlation coefficient.

### Correlations between MvD and various parameters

**Figure 2:**
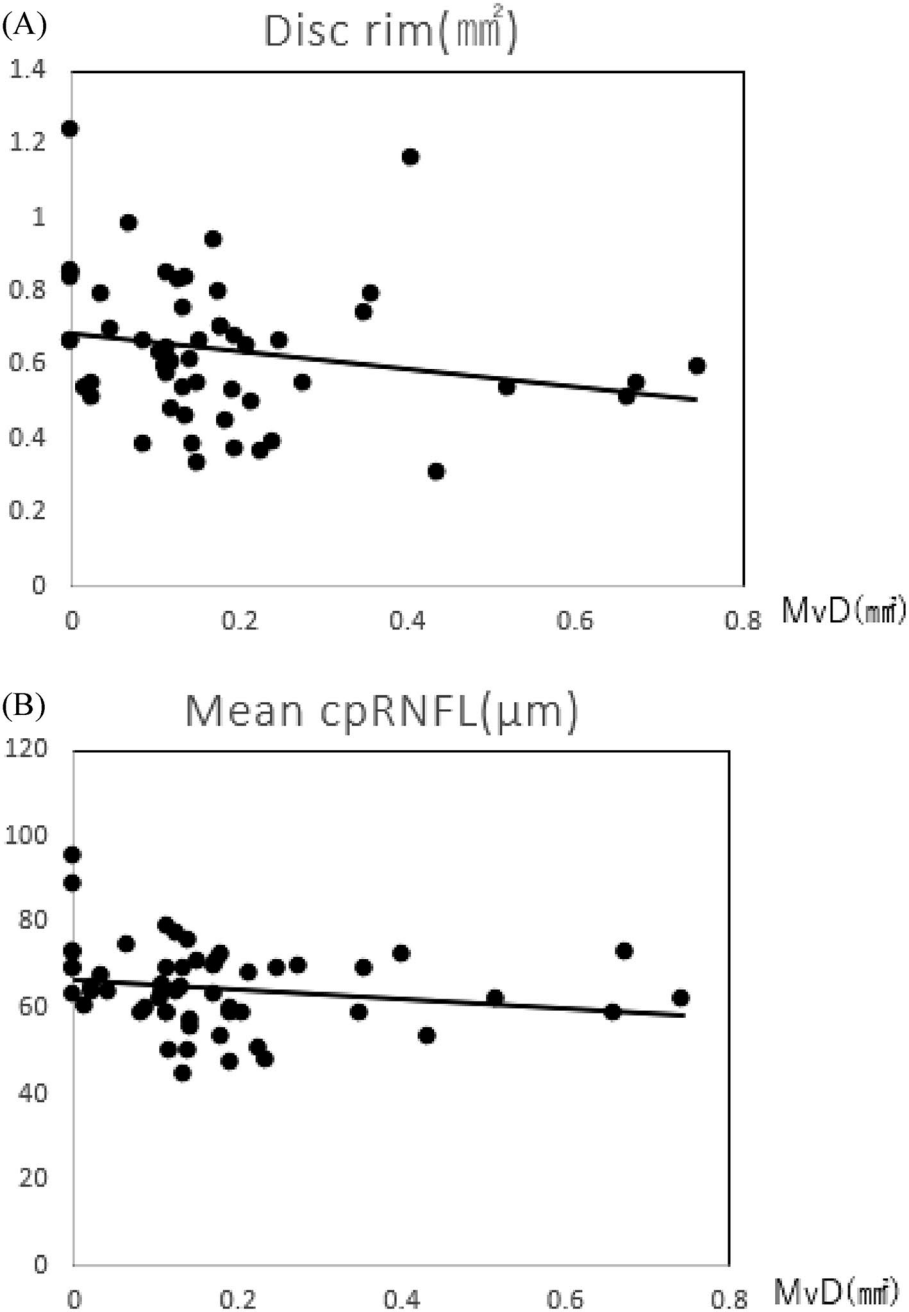
Scatter plots showing correlations between microvasculature dropout and structural indices. The graphs show correlations between microvasculature dropout (MvD) area measured with optical coherence tomography angiography (OCTA) and rim area (**A**) and mean cpRNFL thickness (**B**), respectively. Spearman’s rank correlation coefficient analysis was used to assess correlations.

**Figure 3:**
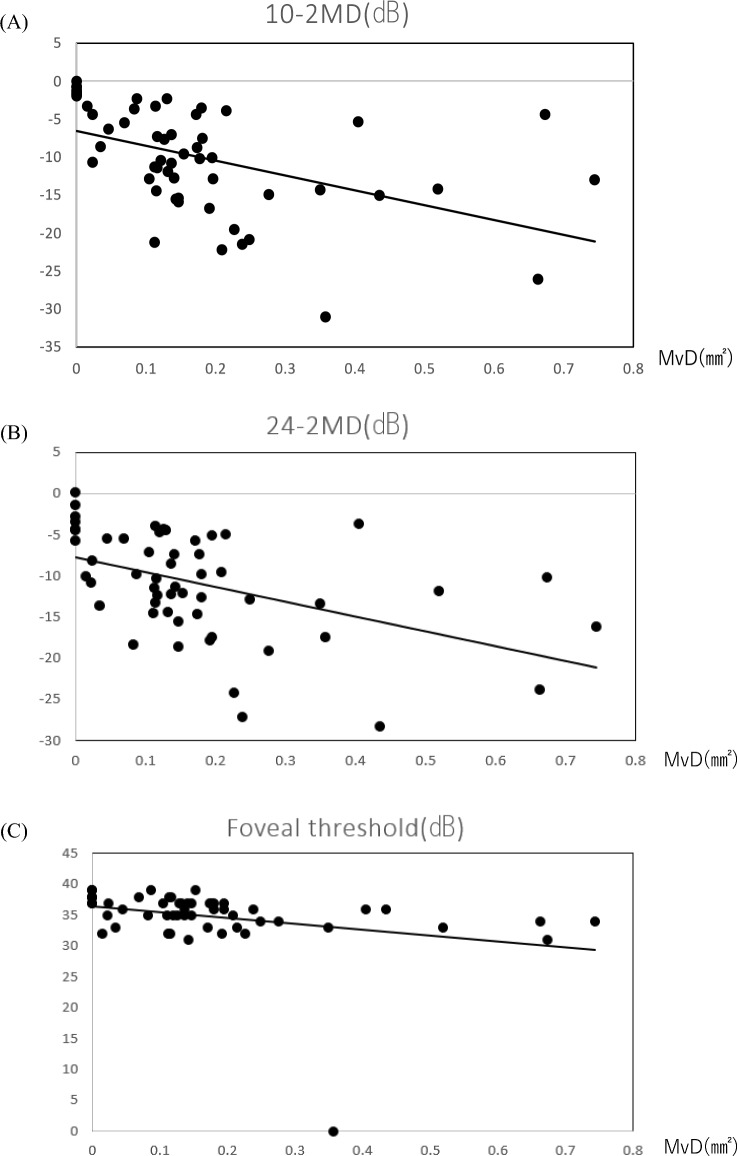
Scatter plots showing correlations between microvasculature dropout and functional indices. The graphs show correlations between microvasculature dropout (MvD) area measured using optical coherence tomography angiography (OCTA) and HFA10-2 MD (**A**), HFA24-2 MD (**B**), and foveal threshold (**C**), respectively. Spearman’s rank correlation coefficient analysis was used to assess correlations.

MvD area, functional indices, structural indices, clinical characteristics, and their relationships are shown in Table 3. MvD area was 0.18 ± 0.17 mm^2^; 87.2% of all patients had findings of MvD. The overall mean optic disc rim area was 0.64 ± 0.19 µm. Mean cpRNFL thickness was 64.62 ± 9.68 µm. The overall foveal threshold (FT) was 35.36 ± 6.50 dB. Mean HFA10-2 MD was − 10.03 ± 7.22 dB and mean HFA24-2 MD was − 10.96 ± 6.43 dB. MvD area was significantly correlated with overall mean optic disc rim area (*p* = 0.01), mean cpRNFL thickness (*p* = 0.02), FT (*p* = 0.005), HFA10-2 MD (*p* < 0.0001), HFA24-2 MD (*p* < 0.0001), HFA10-2 PSD (*p* < 0.0001), and HFA24-2 PSD (*p* < 0.0001). Scatter plots show significant correlations between MvD area and structural indices (optic disc rim area and cpRNFL thickness) and functional indices (HFA10-2 MD, 24-2 MD, and FT) (Figs. 2 and 3).

**Table 3:** Relationship between the presence of MvD and various parameters.

	Total 55 eyes	MvD (+) 48 eyes	MvD (−) 7 eyes	*p*
Age (years)	60.69 ± 12.29	62.25 ± 11.48	50.00 ± 13.18	0.050*
SE (D)	− 3.91 ± 2.99	− 3.78 ± 3.11	− 4.79 ± 1.89	0.258
Axial length (mm)	25.29 ± 1.39	25.21 ± 1.4	25.91 ± 1.23	0.240
CCT (μm)	524.15 ± 38.19	522.3 ± 37.28	536.57 ± 44.96	0.449
PPA area (mm^2^)	1.13 ± 0.72	1.15 ± 0.76	0.99 ± 0.43	0.416
MvD area (mm^2^)	0.18 ± 0.17	0.20 ± 0.17	0.00 ± 0.00	< 0.0001***
Optic disc rim area (mm^2^)	0.64 ± 0.19	0.61 ± 0.18	0.85 ± 0.19	0.017*
Optic disc area (mm^2^)	1.89 ± 0.4	1.88 ± 0.42	1.92 ± 0.25	0.718
Optic disc cupping volume (mm^3^)	0.54 ± 0.24	0.55 ± 0.24	0.45 ± 0.23	0.317
CpRNFL thickness (μm)	64.62 ± 9.68	62.96 ± 8.21	76 ± 11.93	0.027*
10–2 MD (dB)	− 10.03 ± 7.03	− 11.34 ± 6.57	− 1.08 ± 0.65	< 0.0001***
10–2 PSD	10.21 ± 4.64	11.2 ± 3.98	3.47 ± 2.99	< 0.0001***
24–2 MD (dB)	− 10.96 ± 6.43	− 12.1 ± 6.05	− 3.13 ± 1.97	< 0.0001***
24–2 PSD	11.46 ± 3.23	12.17 ± 2.72	6.61 ± 2.11	< 0.0001***
Foveal threshold (dB)	35.36 ± 6.5	34.98 ± 6.87	38.00 ± 0.82	0.005***
IOP (mmHg)	14.18 ± 2.48	13.98 ± 2.5	15.56 ± 1.88	0.078
Decimal VA	1.12 ± 0.2	1.10 ± 0.21	1.20 ± 0.01	0.003**
logMAR	− 0.03 ± 0.16	− 0.02 ± 0.17	− 0.08 ± 0.01	0.028

Unpaired t-test.

*SE* spherical error; *CCT* central corneal thickness; *PPA* peripapillary choroidal atrophy; *MvD* microvasculature dropout; *MD* mean deviation; *PSD* pattern standard deviation; *cpRNFL* circumpapillary retinal nerve fiber layer; *IOP* intraocular pressure; *VA* visual acuity; *logMAR* logarithm of Minimum Angle of Resolution.

*p < 0.05, **p < 0.01, ***p < 0.001.

Clinical characteristics and their relationships with MvD area are shown in Table 3. Significant differences were only observed between MvD area and decimal visual acuity (VA) and between MvD area and the logarithm of the minimum angle of resolution (logMAR) VA.

Table 4 shows the results of multivariable regression analysis with MvD area as the dependent variable and age, mean axial length, IOP, mean cpRNFL thickness, HFA10-2 MD, and PPA area as independent variables.

**Table 4:** Associations with MvD area in multivatriable regression analysis.

Independent variable	B	R^2^ = 0.425	*p* = 0.001	*p*
		SE (B)	β	
Age	0.002	0.002	0.146	0.360
Axial length	0.017	0.017	0.153	0.318
IOP	− 0.010	0.007	− 0.167	0.181
CpRNFL thickness	0.000	0.002	− 0.007	0.956
10–2 MD (dB)	− 0.011	0.003	− 0.492	0.001***
PPA area (mm^2^)	0.072	0.026	0.347	0.008**

MvD, microvasculature dropout; IOP, intraocular pressure; cpRNFL, circumpapillary retinal nerve fiber layer; MD, mean deviation; PPA, peripapillary choroidal atrophy.

** p < 0.01, *** p < 0.001.

Multivariable regression analysis using the simultaneous forced entry method revealed that MvD area is significantly associated with HFA10-2 MD (*p* = 0.001) and PPA area (*p* = 0.008).

## Discussion

In this study, 87.2% of patients (48 of 55 eyes) had MvD. In a similar study using Avanti-XR by our department, 59% of patients (31 of 61 eyes) had MvD. Although the MvD detection rate was higher than in previous reports^23^, there might have been differences in patient characteristics. It is necessary to make a comparative study of patients with similar characteristics in the future. A recent study reported that MvD area and angular circumference change over time and are associated with concurrent cpRNFL loss in eyes with POAG^24^. Thus, it is possible that the patient population in this study included patients with severe disease.

In patients with MvD, functional indices (HFA24-2 MD, HFA10-2 MD, and average overall threshold sensitivity) and morphological indices (PPA area, thickness of each inner layer of the retina, and cpRNFL) were significantly lower than in patients without MvD. Furthermore, regarding clinical characteristics, there was a tendency towards older age and poorer eyesight; thus, it is possible that the finding of MvD is a characteristic of advanced glaucoma.

However, it is unclear whether MvD occurs because glaucoma has progressed or whether glaucoma progresses because of MvD. Recent prospective studies have shown that MvD is a predictor of progressive RNFL thinning in POAG, suggesting that MvD might have progressed earlier^12^.

MvD area was positively correlated with PPA area and negatively correlated with macular inner layer thickness, cpRNFL thickness, HFA24-2 MD, HFA10-2 MD, and visual field sensitivity threshold. Thus, MvD area might be related to structural changes around the optic disc as well as the structure of the inner layer of the retina, which might reflect the severity of glaucoma.

In a previous report, MvD was observed in PPA. PPA area and glaucoma severity were associated with the presence of MvD^2^. Therefore, circulation in the deep microvessels around the papilla is associated with glaucoma optic neuropathy.

This study had some limitations. Regarding MvD measurement, axial length and ocular refraction values were not corrected. Instead, we defined a population of extreme eye axes and reflex values as exclusion criteria from the beginning. We thought that no corrections that would significantly change the results were necessary. In addition, in multivariate analysis, eye axial length was corrected and analyzed. For these reasons, MvD and PPA areas were displayed in units of mm^2^ based on software measurements instead of pixel display, which might have affected accuracy.

## Data Availability

Data supporting the findings of the current study are available from the corresponding author upon reasonable request.
