# Author Correction: Parapapillary choroidal microvasculature dropout in eyes with primary open-angle glaucoma

**DOI:** 10.1038/s41598-024-54133-6

**Published:** 2024-02-19

**Authors:** Ryoko Igarashi, Shun Ochiai, Tadamichi Akagi, Daiki Miyamoto, Yuta Sakaue, Ryu Iikawa, Takeo Fukuchi

**Affiliations:** Department of Ophthalmology and Visual Science, Graduate School of Medical and Dental Sciences, Niigata University, 1-757 Asahimachido-Ri, Chuo-Ku, Niigata, 951-8510 Japan

Correction to: *Scientific Reports* 10.1038/s41598-023-48102-8, published online 23 November 2023

The original version of this Article contained an error in Figure 3(A), where the axis label was incorrect.

The original Figure 3 and accompanying legend appear below.

**Figure 3:**
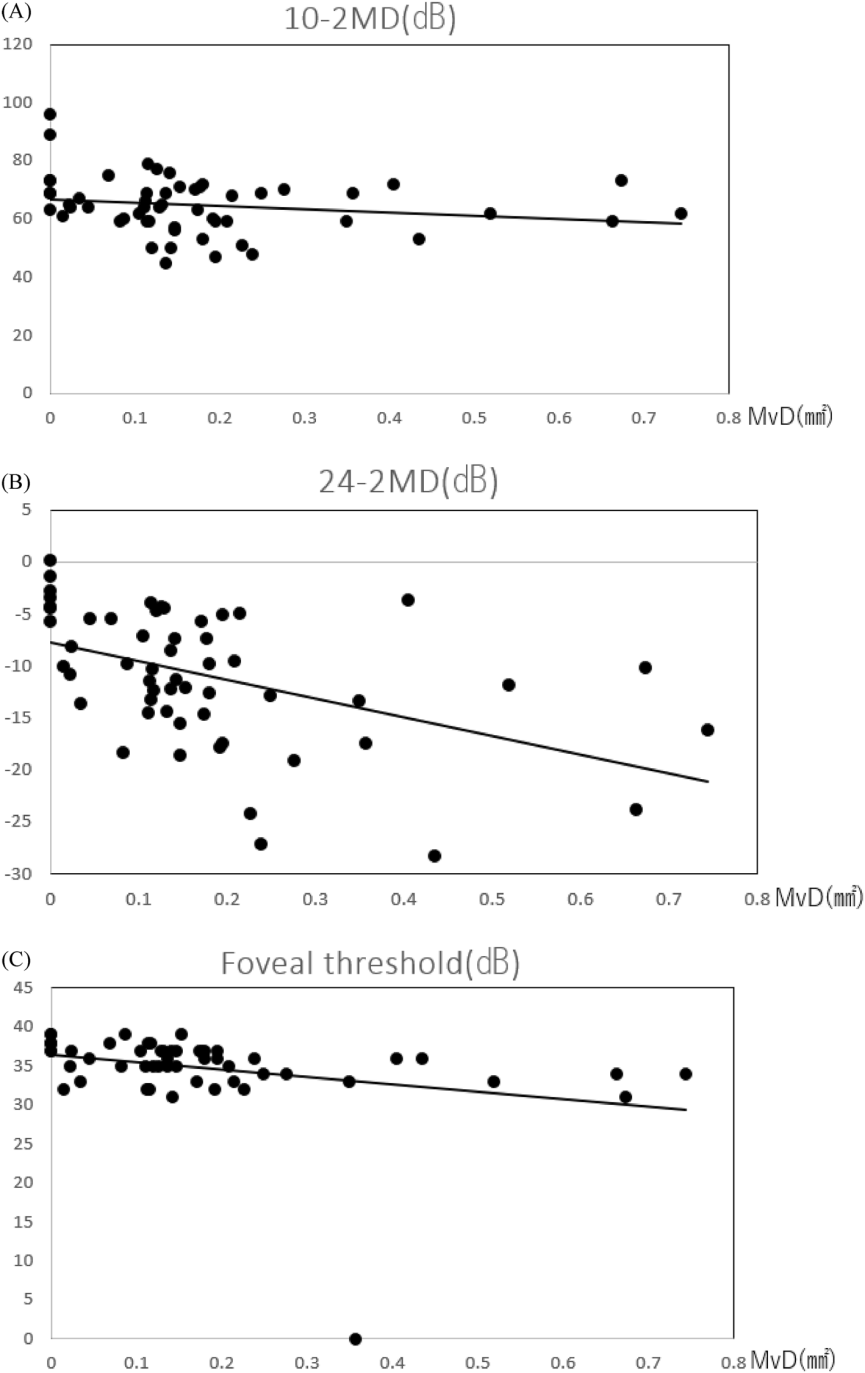
Scatter plots showing correlation between MvD and functional indices (**A**) 10-2 MD (**B**) 24-2 MD (**C**) Foveal threshold.

The original Article has been corrected.

